# Naringenin Attenuates Isoprenaline-Induced Cardiac Hypertrophy by Suppressing Oxidative Stress through the AMPK/NOX2/MAPK Signaling Pathway

**DOI:** 10.3390/nu15061340

**Published:** 2023-03-09

**Authors:** Yu Li, Bo He, Chao Zhang, Yanji He, Tianyang Xia, Chunyu Zeng

**Affiliations:** 1 Department of Cardiology, Daping Hospital, Third Military Medical University (Army Medical University), Chongqing 400042, China; 2 Chongqing Key Laboratory for Hypertension Research, Chongqing Cardiovascular Clinical Research Center, Chongqing Institute of Cardiology, Chongqing 400042, China; 3 State Key Laboratory of Trauma, Burns and Combined Injury, Daping Hospital, Third Military Medical University (Army Medical University), Chongqing 400042, China; 4 Cardiovascular Research Center of Chongqing College, University of Chinese Academy of Sciences, Chongqing 400042, China

**Keywords:** naringenin, cardiac hypertrophy, oxidative stress, AMPK, NOX2

## Abstract

Cardiac hypertrophy is accompanied by increased myocardial oxidative stress, and whether naringenin, a natural antioxidant, is effective in the therapy of cardiac hypertrophy remains unknown. In the present study, different dosage regimens (25, 50, and 100 mg/kg/d for three weeks) of naringenin (NAR) were orally gavaged in an isoprenaline (ISO) (7.5mg/kg)-induced cardiac hypertrophic C57BL/6J mouse model. The administration of ISO led to significant cardiac hypertrophy, which was alleviated by pretreatment with naringenin in both in vivo and in vitro experiments. Naringenin inhibited ISO-induced oxidative stress, as demonstrated by the increased SOD activity, decreased MDA level and NOX2 expression, and inhibited MAPK signaling. Meanwhile, after the pretreatment with compound C (a selective AMPK inhibitor), the anti-hypertrophic and anti-oxidative stress effects of naringenin were blocked, suggesting the protective effect of naringenin on cardiac hypertrophy. Our present study indicated that naringenin attenuated ISO-induced cardiac hypertrophy by regulating the AMPK/NOX2/MAPK signaling pathway.

## 1. Introduction

Cardiac hypertrophy is a compensatory response to environmental stimuli, and it can be classified as physiologic and pathologic hypertrophy [1,2]. Cardiac hypertrophy involves multiple mechanisms such as long-term hemodynamic load, neurohormonal stimuli and cardiomyocyte metabolism. Perpetuated cardiac hypertrophy aggravates heart failure, affecting about 64.3 million people worldwide [3]. Currently, limited therapeutic options are available for patients with cardiac hypertrophy. Therefore, an effective therapeutic intervention for cardiac hypertrophy is urgently needed. Increased myocardial oxidative stress is an essential factor contributing to cardiac hypertrophy [4]. Under physiological conditions, reactive oxygen species (ROS) production and elimination are in a dynamic balance. However, oxidative stress occurs once the balance is broken [5]. Increased oxidative stress damages proteins, lipids, DNA, and RNA [6]. Meanwhile, excessive ROS significantly activates multiple hypertrophy-related genes, including NF-KB and NRF2 [5,7]. Therefore, antioxidant therapy has been proposed as an effective treatment for cardiac hypertrophy. However, the general antioxidant drugs, including vitamins C and E, do not yield satisfactory results in clinical experiments [8,9], whereas ROS targeted strategies and modulated downstream pathways have been proven to be better approaches. Hence, the exploration of new antioxidants is helpful in preventing cardiac hypertrophy.

Naringenin is a natural flavanone found in citrus fruits such as oranges and shows many biological activities [10]. Naringenin appears in nature in two primary forms: aglycosylated (naringenin) and glycosylated (naringin or naringenin-7-O-glucoside) [11]. Flavonoids are widely found in fruits and vegetables and have been proven to alleviate cardiovascular disease, primarily due to their antioxidant properties. Epidemiological and prospective studies suggest that naringenin, one of the essential flavonoids, has beneficial effects on increasing cardiovascular risk [12]. The therapeutic effects of naringenin have been proven in several diseases due to its antioxidant, anti-inflammation, antiapoptotic, and antitumor activities, including chronic kidney disease, cancer, neurological disorders, and liver disease [13,14,15]. The pretreatment of naringenin before ischemia-reperfusion injury in the isolated hearts of rats effectively improved the systolic function of the left ventricle by activating the ATP-sensitive potassium channels on the cell membrane [16]. Naringenin also acts as an NADPH oxidase inhibitor. Naringenin alleviates diabetic nephropathy and high-cholesterol diet-induced endothelial dysfunction by inhibiting NADPH oxidase 4 (NOX4) and NOX2 [17]. In cardiovascular diseases, naringenin decreases NADPH oxidase activity, inhibits oxidative stress, and migrates hyperglycemia-induced myocardial fibrosis. Additionally, naringenin treatment inhibits the PI3K/Akt, ERK, and JNK signaling pathways to alleviate pressure overload-induced cardiac hypertrophy [18]. It has been noted that naringenin reduces LDL, increases HDL, inhibits macrophage inflammation, inhibits foam cell formation, and significantly down-regulates the expression of genes related to atherosclerosis, thus having a significant anti-atherosclerosis effect [19]. Due to insufficient data on pharmacokinetics, metabolic fate, and chemical instability, clinical trial registration is insufficient. Therefore, in this research, we evaluate the therapeutic effects of naringenin on isoprenaline (ISO)-induced cardiac hypertrophy induced by the continuous stimulation of β-adrenergic receptors, and determine the underlying mechanism relating to the inhibition of oxidative stress. 

## 2. Materials and Methods

### 2.1. Animals

All the mouse procedures were performed according to the Institutional Animal Care and Use Committee of the Army Medical University (Animal Ethical Statement AMUWEC20226269). C57BL/6J mice (at age 8–10 weeks) were housed in the animal center at a temperature of 18–22 °C with free access to food and water. Naringenin was suspended in carboxy methyl-cellulose (0.7%). Mice in the control group were then administered 0.7% carboxy methyl-cellulose via gavage. To induce cardiac hypertrophy, mice of either sex were treated with ISO (7.5 mg/kg/d, MedChemExpress, Princeton, NJ, USA) by subcutaneous injection for 2 weeks [20]. To determine the role of naringenin (NAR) in cardiac hypertrophy, naringenin was given by means of gavage administration with different dosage regimens (25, 50 and 100 mg/kg/d, Aladdin, Shanghai, China) for 1 week and then co-administration with ISO for another 2 weeks. The scheme of the experimental design is shown in Supplementary Figure S1.

### 2.2. Echocardiography

The acquisition of echocardiographic images was performed through a Vevo 3100LT system (VisualSonics, Fujifilm, Tokyo, Japan), and the analysis of echocardiographic images was conducted by using Vevo LAB 3.1.0 software. The mice were anesthetized in an induction box by inhaling 2% isoflurane and kept anesthetized at a lower concentration. Additionally, the heart rate of mice was controlled at 400–450 bpm. The representative M-mode images were captured, and M-mode tracings were used to measure end-diastolic and end-systolic left ventricular inner diameters (LVIDd, LVIDs). Left ventricular ejection fraction (EF) and fractional shortening (FS) were also obtained from the Vevo system. The operation and measurement of echocardiograph data were performed by two experienced echocardiographers blinded to the study design. 

### 2.3. Cell Isolation and Culture

As previously reported, the isolation of neonatal rat cardiomyocytes (NRCMs) was performed [21]. Briefly, neonatal rats were intraperitoneally injected with heparin and later anesthetized with isoflurane. The hearts were excised and fastened onto a Langendorff system. Subsequently, the harvested hearts were perfused with a pre-cooled medium for 8 min to remove as much blood as possible. The perfusion medium consisted of the following ingredients: 4 mM KCl, 140 mM NaCl, 1 mM MgCl_2,_ 10 mM taurine, 10 mM HEPES, 10 mM glucose, and 10 mM 2,3-butanedione monoxime, with the medium’s pH adjusted to 7.3. Later the perfusion medium was replaced with a digestion buffer, which continued to infuse the heart for 15 min until the samples became soft. The digestion buffer was prepared by adding 0.12 mg/mL trypsin, 1 mg/mL collagenase II, and 0.02 mM CaCl_2_ to the perfusion buffer. Then, the hearts were transferred to a new culture dish with pre-cooled PBS solution, trimmed into smaller pieces, and moved into a cell suspension. The stop buffer was prepared by adding 0.1 mM CaCl_2_ to the perfusion buffer and terminating digestion with 5 mg/mL bovine serum albumin. The cells in the suspension were filtered with a 75 μm cell strainer, collected in a sterile centrifuge tube, centrifuged at 100× *g* for 5 min, and resuspended in DMEM medium containing 10% FBS. Two hours later, non-adherent cells were removed, collected in a new sterile centrifuge tube, and centrifuged at 100× *g* for 5 min. Then, the supernatant was removed, and the non-adherent cardiomyocytes were resuspended, counted, and seeded into sterile plates for further study. To examine the cell viability of NRCMs after being treated with naringenin at different concentrations for 24 h, the cells were incubated with CCK8 solution (Beyotime, Shanghai, China), and their absorbance values were measured at 570 nm. 

### 2.4. Masson Trichrome Staining and Immunofluorescence Staining

After fixation, dehydration, and embedding, the heart tissues were sectioned at a thickness of 5 μm. Masson-trichrome staining was performed following the instructions provided with the Masson-trichrome staining kit (Solarbio, Beijing, China) to evaluate cardiac fibrosis. The areas where fibrosis occurred were stained blue. The proportion of fibrosis was calculated as the ratio of the blue area to the total area. Cell membrane staining was performed using Oregon Green 488 conjugated WGA (Invitrogen, Carlsbad, CA, USA) to assess the cross-sectional area of cardiomyocytes and cardiac hypertrophy. The sections were incubated with dilute WGA solution at 37 °C in the dark for 45 min, and later washed with PBS solution three times (each time for 5 min) and stained with DAPI for 2 min. cTnT staining was used to identify the isolated NRCMS and the hypertrophy of NRCMs induced by ISO via observing the size of the cell area. In brief, after washing with PBS solution, the treated cells were fixed in 4% paraformaldehyde solution at room temperature for 10 min. After washing with PBS solution three times (each time for 5 min), the cells were incubated with the blocking solution for 30 min at room temperature and later incubated with diluted cTnT antibody solution at 4 °C overnight. The following day, the cells were washed with PBS solution three times (each time for 5 min), then incubated with the donkey anti-mouse IgG *Alexa Fluor*^®^488 (Invitrogen, Carlsbad, USA) at 37 °C for 1 h. Before the images were captured, the cells were washed with PBS solution three times (each time for 5 min), and stained with DAPI for 2 min. Images were obtained with a Zei ss LSM 880 upright confocal fluorescence microscope. The image results were analyzed using Fiji software (Image J version 1.52i). 

### 2.5. Oxidative Stress Detection

Dihydroethidium (DHE) staining was used to analyze ROS generation. The live cells or fresh frozen myocardial sections were incubated with DHE at 37 °C for 40 min. Subsequently, the representative fluorescence images were captured using a digital camera (Olympus DP80, Tokyo, Japan). Following the manufacturer’s instructions, the MDA level and SOD activity were measured with ELISA kits purchased from the Jiancheng Bioengineering Institute (Nanjing, China). 

### 2.6. Real-Time Quantitative PCR Analysis

Following the manufacturer’s instructions, total RNA was extracted from the heart tissues or cell samples with Trizol reagent. Then, the extracted RNA was reversely transcribed into cDNA according to the requirements of the reverse transcription kit (Takara Biotechnology, Dalian, China). The relative RNA expression level of target genes was quantified in each sample using Syber green-based quantitative PCR. Two wells were set up in each DNA sample, and the values were averaged. The PCR primers used are listed in the Supplementary Table S1. The relative expressions of the genes were calculated using the 2^−ΔΔCT^ method. Additionally, the final data are represented as the fold change referring to 2^−ΔΔCT^ treated/2^−ΔΔCT^ control. 

### 2.7. Western Blot Analysis

Total protein was extracted from the heart tissues or cell samples using RIPA lysis buffer (Beyotime, Shanghai, China). In order to detect the expression of phosphorylated proteins, phosphatase inhibitors were also added to the protein extraction process. The total protein concentration in each sample was detected using a BCA protein assay kit (Beyotime, Shanghai, China). A suitable loading buffer was added to the protein according to the measured protein concentration, and the mixtures were denatured at 100 °C. The prepared protein was separated through SDS-polyacrylamide gel electrophoresis (SDS-PAGE) and later transferred to the PVDF membranes. After incubating with a quick blocking solution for 30 min at room temperature, the membranes were incubated with different primary antibodies diluted in a diluent blocking solution at 4 °C overnight. The following day, after washing the membrane three times with TBST (each time for 15 min), the membranes were incubated with appropriate fluorescent second antibodies in the dark for 1 h at room temperature. Then, the results were examined and analyzed with the help of the Odyssey Infrared Imaging System (Li-Cor Biosciences, Lincoln, NE, USA) and software. The primary antibodies included phospho-P38, total-P38, phospho-JNK, total-JNK, phospho-ERK, total-ERK, and GAPDH (Proteintech, Wuhan, China). 

### 2.8. Statistical Analysis

All of the acquired numeric data are presented as the mean ± standard error (mean ± SE). Statistical analyses were performed using *SPSS 21.0* software. Comparisons between multiple groups were performed using a one-way analysis of variance followed by Tukey’s multiple comparisons. Additionally, the comparisons between the two groups were analyzed using Student’s *t*-test. *p* < 0.05 was considered statistically significant. 

## 3. Results

### 3.1. Naringenin Attenuated Isoprenaline (ISO)-Induced Cardiac Hypertrophy

**Figure 1:**
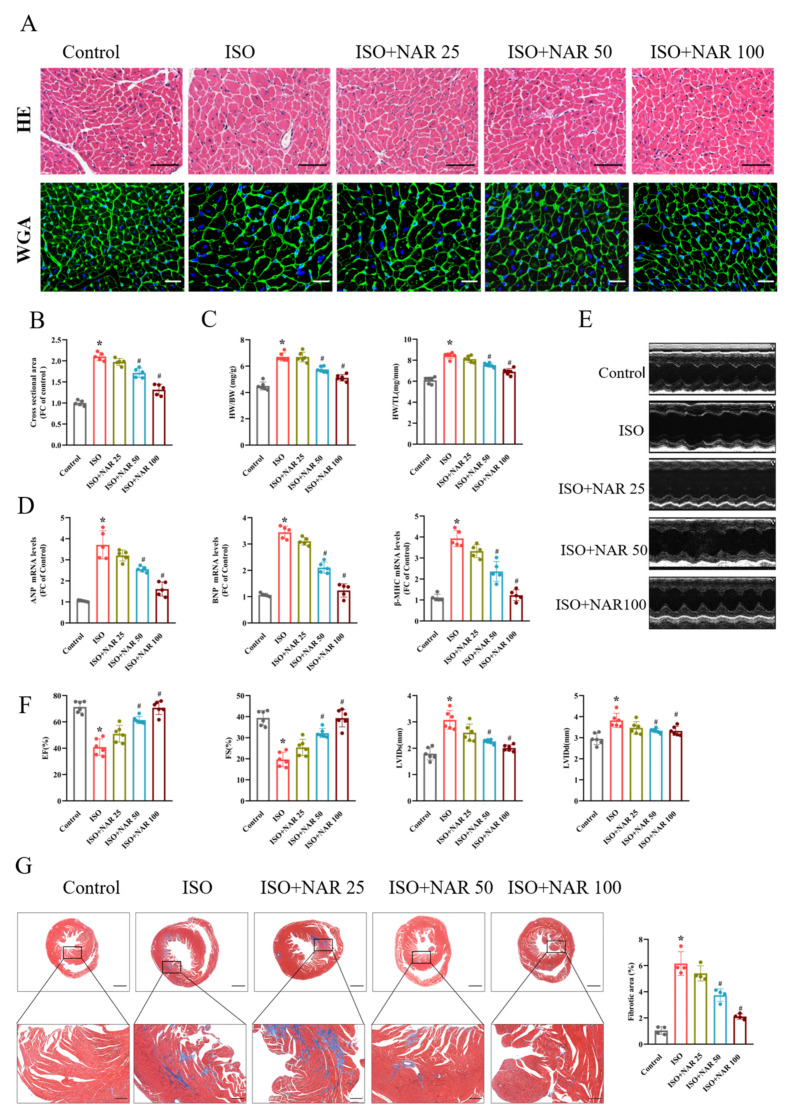
Naringenin attenuated isoprenaline-induced cardiac hypertrophy in C57BL/6J mice with or without different dosages (25, 50, and 100 mg/kg/d) of naringenin (NAR) for three weeks and then co-treatment with isoprenaline (ISO, 7.5mg/kg/d) by subcutaneous injection from the second week. (**A**) Representative HE and wheat germ agglutinin (WGA) staining images in C57BL/6J mice cardiac tissues. The scale bars in the HE and WGA images are 100 μm and 20 μm, respectively. (**B**) Quantification of cardiomyocyte from cross-sectional areas of WGA staining (n = 5 slides with 10 fields/slide). (**C**) Ratio of heart weight/body weight (HW/BW) and heart weight/tibial length (HW/TL) of mice that received NAR pretreatment (n = 6). (**D**) The mRNA expressions of hypertrophy markers, including ANP, BNP, and β-MHC, quantified through qRT-PCR (n = 5). (**E**) Representative M-mode echocardiographic images. (**F**) The echocardiographic parameters, including left ventricular ejection fraction (EF), fractional shortening (FS), and end-diastolic and end-systolic left ventricular inner diameters (LVIDd, LVIDs) (n = 6). (**G**) Representative images of Masson staining and analysis of cardiac fibrosis area (n = 4). The scale bars are 1 mm (upper panel) and 200 μm (lower panel), respectively. * *p* < 0.05 versus the control group and ^#^ *p* < 0.05 versus the ISO group.

Consistent with previous studies, ISO subcutaneous injection (7.5 mg/kg/d for 2 weeks) significantly induced pathological cardiac hypertrophy, as demonstrated by the increased cardiomyocyte cross-sectional area demonstrated in the HE and WGA staining results (Figure 1A,B), the increased ratio of heart weight/body weight (HW/BW) and heart weight/tibial length (HW/TL) (Figure 1C), and the increased expression of hypertrophic marker genes (ANP, BNP, and β-MHC) (Figure 1D), which were dose-dependently (ranging from 25 to 100 mg/kg/d) reduced by pretreatment with gavage-administrated naringenin (Figure 1A–D). We also investigated cardiac function through echocardiographic analysis. In ISO-induced cardiac hypertrophy mice, cardiac dysfunction was reflected by decreased EF and FS, and increased LVIDs and LVIDd (Figure 1E,F). However, naringenin pretreatment significantly attenuated ISO-induced cardiac dysfunction. Meanwhile, the results from Masson staining showed that naringenin pretreatment reduced the cardiac fibrotic area in ISO-induced hypertrophied hearts (Figure 1G). 

### 3.2. Naringenin Ameliorated ISO-Induced Cardiomyocyte Hypertrophy by Inhibiting Oxidative Stress through AMPK/NOX2/MAPK Signaling Pathway

**Figure 2:**
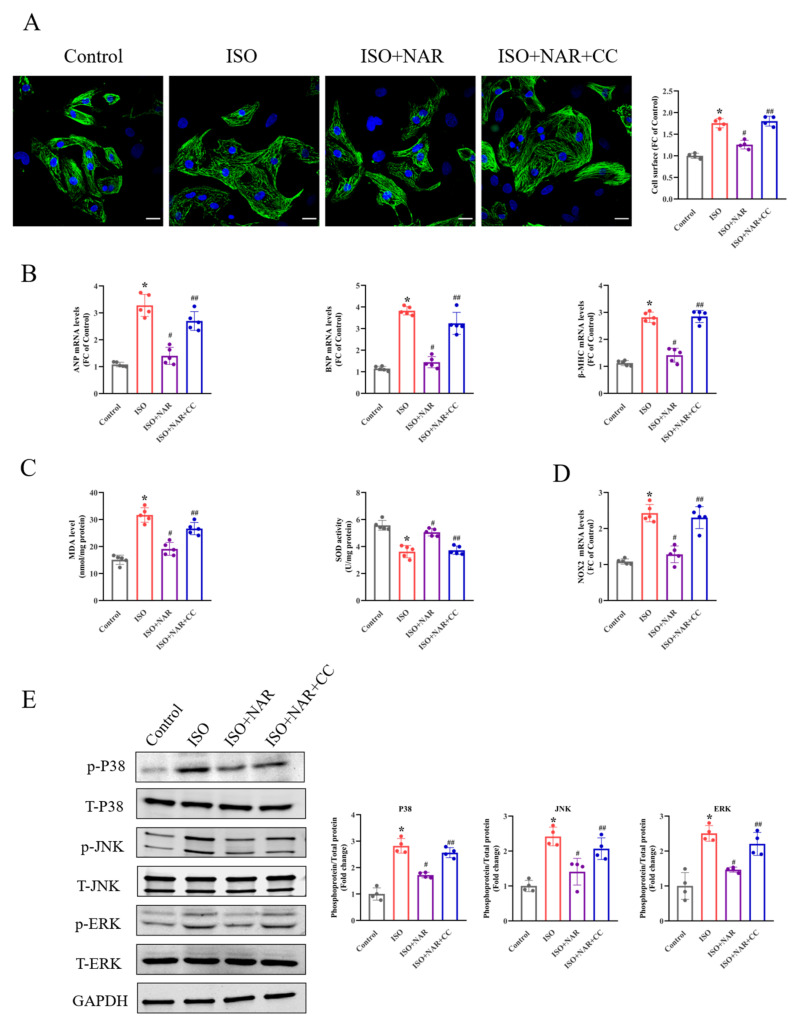
Naringenin ameliorates ISO-induced cardiomyocyte hypertrophy by inhibiting oxidative stress through the AMPK/NOX2/MAPK signaling pathway. (**A**) Representative images of cTnT-positive NRCMs and the quantification of cell surface area of NRCMs (n = 4 slides with 10 fields/slide). The scale bars represent 20 μM. (**B**) The expressions of ANP, BNP, and β-MHC, detected through qRT-PCR (n = 5). (**C**) The MDA levels and SOD activity of NRCMs (n = 5). (**D**) The expression of NOX2 in NRCMs, detected through qRT-PCR (n = 5). (**E**) The protein expressions of p-P38, p-JNK, and p-ERK in NRCMs, detected through Western blots (n = 4). * *p* < 0.05 versus the control group, ^#^ *p* < 0.05 versus the ISO group, and ^##^ *p* < 0.05 versus the ISO+NAR group. CC, compound C.

Cell viability was assessed at different concentrations of naringenin to further determine the effect of naringenin on cardiomyocytes (Supplementary Figure S2). The anti-hypertrophic effect of naringenin was confirmed in vitro. ISO treatment (10 μM, 24 h) notably increased cell sizes and the expression of hypertrophic markers (ANP, BNP, and β-MHC) in NRCMs, which were attenuated by naringenin treatment (10 μM) (Figure 2A,B). 

It is reported that oxidative stress is essential in ISO-induced cardiac hypertrophy [22]. As expected, the MDA level in ISO-induced hypertrophied hearts was significantly increased, while the SOD activity was decreased. The pretreatment of naringenin effectively inhibited ISO-induced oxidative stress, as demonstrated by reduced MDA level and increased SOD activity (Figure 2C). The primary source of ROS in the heart is NADPH oxidase [18]. Of the NADPH oxidase isoforms expressed in the heart (NOX2 and NOX4), NOX2 may be responsible for β-AR agonist-induced cardiac hypertrophy [23,24]. The naringenin pretreatment significantly inhibited the elevation of NOX2 in ISO-induced hypertrophic NRCMs (Figure 2D). Previous studies demonstrated that the increased ROS generated by ISO treatment stimulated the activation of the MAPK signaling pathway [25]. Consistent with these results, our study found that ISO significantly stimulated the phosphorylation of P38, JNK, and ERK. On the other hand, naringenin inhibited the activation of MAPK signaling and the phosphorylation of P38, JNK, and ERK (Figure 2E). 

AMPK has been recognized as an energy sensor that plays a critical role in maintaining redox balance [26,27]. It has been verified that ISO-induced hypertrophy is accompanied by the inhibition of AMPK [28]. Naringenin has been reported to activate AMPK [29]. Therefore, we further investigated the characteristics of AMPK in the anti-hypertrophic effect of naringenin. We found that the pretreatment of the selective AMPK inhibitor compound C (20 μM, 4 h) almost eliminated the anti-hypertrophic effects of naringenin, as demonstrated by the enlarged cell size and increased expression of hypertrophic markers (Figure 2A,B). Meanwhile, naringenin-induced decreases in oxidative stress and inhibition of MAPK signaling were also mostly eliminated by compound C in NRCMs (Figure 2C–E). These results suggest that the anti-hypertrophic effect of naringenin on ISO-induced hypertrophied NRCMs is attributed to the AMPK/NOX2/MAPK signaling pathway. 

### 3.3. Inhibition of AMPK Blocked the Anti-Hypertrophic Effects of Naringenin on ISO-Induced Cardiac Hypertrophy In Vivo

**Figure 3:**
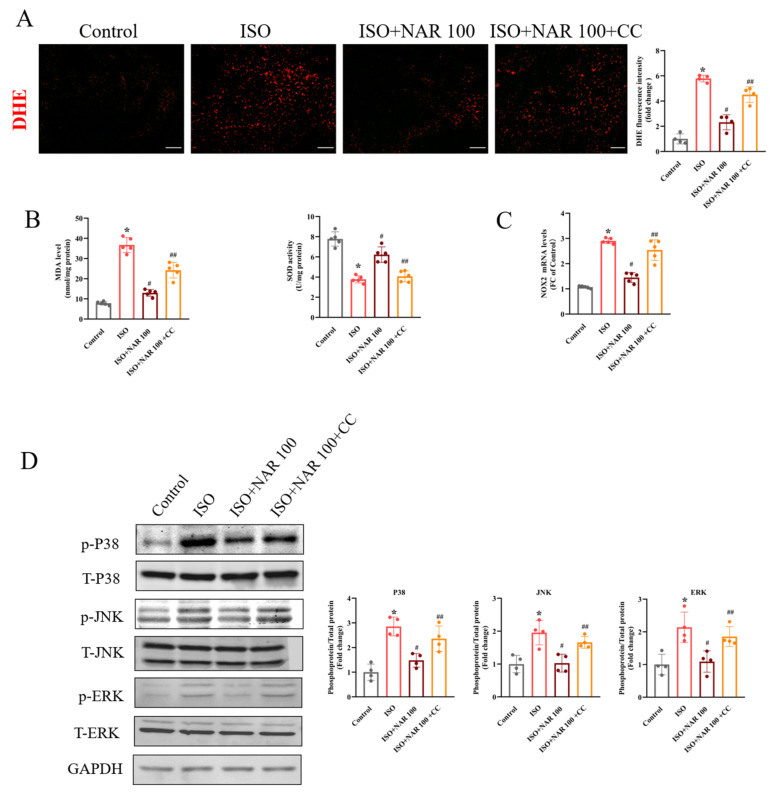
Effect of naringenin on oxidative stress in ISO-induced hypertrophied hearts. (**A**) Representative images of DHE staining and the analysis of DHE fluorescence in heart tissues (n = 4). The scale bars are 20 μm. (**B**) MDA levels and SOD activity in heart (n = 5). (**C**) The expressions of NOX2 in heart tissues, detected through qRT-PCR (n = 5). (**D**) The expressions of p-P38, p-JNK, and p-ERK in heart tissues, detected through Western blots (n = 4). * *p* < 0.05 versus the control group, ^#^ *p* < 0.05 versus the ISO group, and ^##^ *p* < 0.05 versus the ISO+NAR 100 group. CC, compound C.

**Figure 4:**
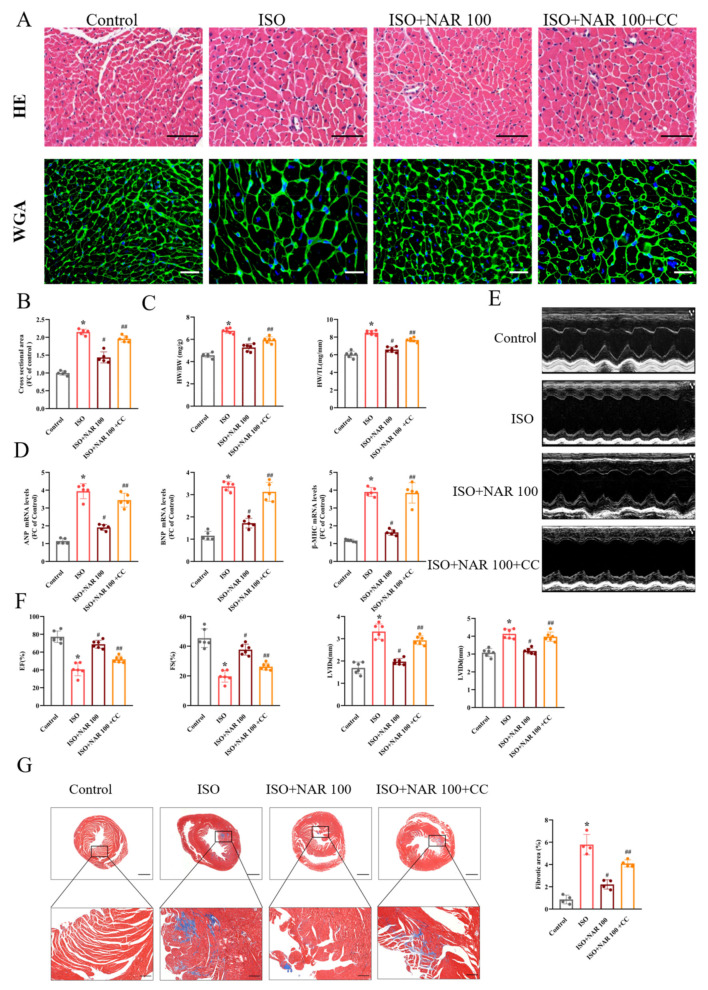
Inhibition of AMPK blocked the anti-hypertrophic effects of naringenin on ISO-induced cardiac hypertrophy in vivo. C57BL/6J mice were given 100 mg/kg/d of NAR via gavage for three weeks, and administrated 7.5 mg/kg/d of ISO via subcutaneous injection with or without compound C (CC, 20 mg/kg) for the next two weeks. (**A**) Representative HE and WGA staining images in C57BL/6J mice heart tissues. The scale bars in HE and WGA images are 100 μm and 20 μm, respectively. (**B**) Quantification results for cardiomyocyte from cross-sectional areas of WGA staining (n = 5 slides with 10 fields/slide). (**C**) Ratio of HW/BW and HW/TL (n = 6). (**D**) The mRNA expressions of hypertrophy markers, including ANP, BNP, and β-MHC, detected through qRT-PCR (n = 5). (**E**) Representative echocardiographic images (M-mode). (**F**) Cardiac function parameters, including EF, FS, LVIDs, and LVIDd, measured through echocardiography (n = 6). (**G**) Representative images of Masson staining and the quantification analysis of the cardiac fibrosis area (n = 4). The scale bars are 1 mm (upper panel) and 200 μm (lower panel), respectively. * *p* < 0.05 versus the control group, ^#^ *p* < 0.05 versus the ISO group, and ^##^ *p* < 0.05 versus the ISO+NAR 100 group. CC, compound C.

Given the anti-oxidative effect of naringenin on ISO-induced cardiomyocyte hypertrophy, the impact of naringenin on oxidative stress in hypertrophic hearts was investigated. We found that in the ISO-induced hypertrophied hearts, the oxidative stress levels were significantly elevated, as demonstrated by the increased ROS production and MDA level and decreased SOD activity (Figure 3A,B). Meanwhile, the expression of NOX2 and the phosphorylation of P38, JNK, and ERK increased in hypertrophied hearts (Figure 3C,D). Naringenin effectively inhibited the levels of oxidative stress and the activation of MAPK; however, the inhibition of AMPK by compound C (20 mg/kg, 2 weeks) significantly blunted the anti-oxidative effects of naringenin (Figure 3A–D). As shown in Figure 4, ISO-induced cardiac hypertrophy was alleviated considerably by naringenin, as demonstrated by the reduced cell size, HW/BW ratio, HW/TL ratio (Figure 4A–C), and expression of hypertrophic marker genes (ANP, BNP, and β-MHC) (Figure 4D). Naringenin also resulted in cardiac function improvement, with improved echocardiography parameters (namely EF, FS, LVIDs, and LVIDd) (Figure 4E,F) and cardiac fibrosis area reduction (Figure 4G). However, the anti-hypertrophic effects of naringenin were mostly diminished by compound C in ISO-induced cardiac hypertrophy mice (Figure 4A–G). These results suggest that the activation of AMPK contributed to the cardioprotective effects of naringenin in ISO-induced cardiac hypertrophy mice.

## 4. Discussion

Pathological cardiac hypertrophy in response to stress stimuli results in cardiac dysfunction, and eventually develops into heart failure [30,31]. Excessive activation of adrenergic receptors is one of the hallmarks of pathological hypertrophy in patients with heart failure, and sustained β-adrenergic receptor stimulation increases mortality in patients [32]. Evidence shows that oxidative stress plays a crucial role in cardiac hypertrophy [4,7]. Substantial studies demonstrated that ROS in cardiac redox signaling is associated with mitochondrial dysfunction and myocardial fuel utilization. In particular, most oxygen expenditure occurs in cardiomyocytes, where redox signaling facilitates its involvement in homeostatic and stress response pathways, physiological processes, and pathological changes. Thus, oxidative stress suppression represents an important therapeutic target for cardiac hypertrophy. However, in numerous clinical trials, conventional antioxidants, including beta carotene and vitamins C and E, were proven to be insufficient to prevent cardiac hypertrophy [33,34,35]. Although the reasons for this are unclear, conventional antioxidants lacking a direct target to reduce ROS generation might be a key point. NADPH oxidases are verified to be the primary source generating ROS in cardiac hypertrophy. Thus, naringenin, a natural antioxidant targeting NADPH oxidase inhibition, is a potential drug with which to suppress cardiac hypertrophy [36,37]. In the present study, our results found that the pretreatment with naringenin effectively alleviated ISO-induced cardiac hypertrophy and cardiac dysfunction in mice, which might be related to inhibiting oxidative stress, suggesting that naringenin may be a novel therapeutic approach for pathological cardiac hypertrophy.

Naringenin is thought to be the predominant element in a diet high in vegetables and fruit for atherosclerosis [19]. The therapeutic effect of naringenin has been proven in several diseases associated with oxidative stress. In this study, cardiac hypertrophy was induced by the stimulation of abnormal neurohumoral factors through isoprenaline administration. The ISO-induced cardiac hypertrophy was assessed using increased cardiomyocyte cross-sectional area from HE and WGA staining, increased expression of cardiac hypertrophy marker genes (ANP, BNP, and β-MHC), and increased heart size determined with increased ratios of HW/BW and HW/TL. Meanwhile, the sustained ISO administration in mice resulted in cardiac dysfunction, mainly with contractile dysfunction, evaluated through echocardiographic examination with the EF, FS, LVIDs, and LVIDd measurements, as well as exaggerated fibrosis assessed using Masson staining. However, the pretreatment with naringenin significantly mitigated the morphological, pathological, and functional changes caused by ISO-induced pathological cardiac hypertrophy. Moreover, the anti-hypertrophic effect was confirmed in the in vitro experiment. Naringenin alleviated the increased cell area and gene expression of cardiac hypertrophy markers in NRCMs pretreated with ISO. Due to the essential role of oxidative stress in ISO-induced cardiac hypertrophy, we detected the level of oxidative stress in the heart tissues and NRCMs after naringenin treatment. We found that naringenin decreased SOD activity and increased MDA levels in both in vivo and in vitro experiments. These results implied that the inhibition of oxidative stress is a possible mechanism of the naringenin-mediated anti-hypertrophic effect.

AMPK, a potent NOX inhibitor, has been proven to be essential in cardiac remodeling, such as cardiac hypertrophy, fibrosis, and inflammation [38]. The activation of AMPK reduced the generation of NOX-derived ROS and further inhibited cardiac hypertrophy [39,40]. The activation of AMPK by naringenin has been demonstrated in cardiovascular diseases [41,42]. Previous studies indicated that naringenin could activate the AMPK-SIRT3 signaling pathway, inhibit mitochondrial oxidative stress damage, and erase cardiac ischemia-reperfusion injury [41]. These studies indicated that the activation of AMPK was strongly implicated in the cardioprotective effect of naringenin in ISO-induced cardiac hypertrophy. Our present study showed that AMPK inhibitor compound C almost eliminated the anti-hypertrophic effects of naringenin, indicating that AMPK is involved in the pathological process. NADPH oxidases are the primary source of ROS. Among the isoforms of NADPH oxidase, NOX2 is closely associated with cardiac hypertrophy induced by the chronic stimulation of β-adrenergic receptors [23,24]. In our study, a remarkable increase in NOX2 expression was observed in NRCMs and hypertrophic hearts after ISO treatment, while naringenin pretreatment significantly reduced the expression of NOX2. These results implied that the therapeutic effect of naringenin on cardiac hypertrophy is related with the AMPK signaling pathway.

**Figure 5:**
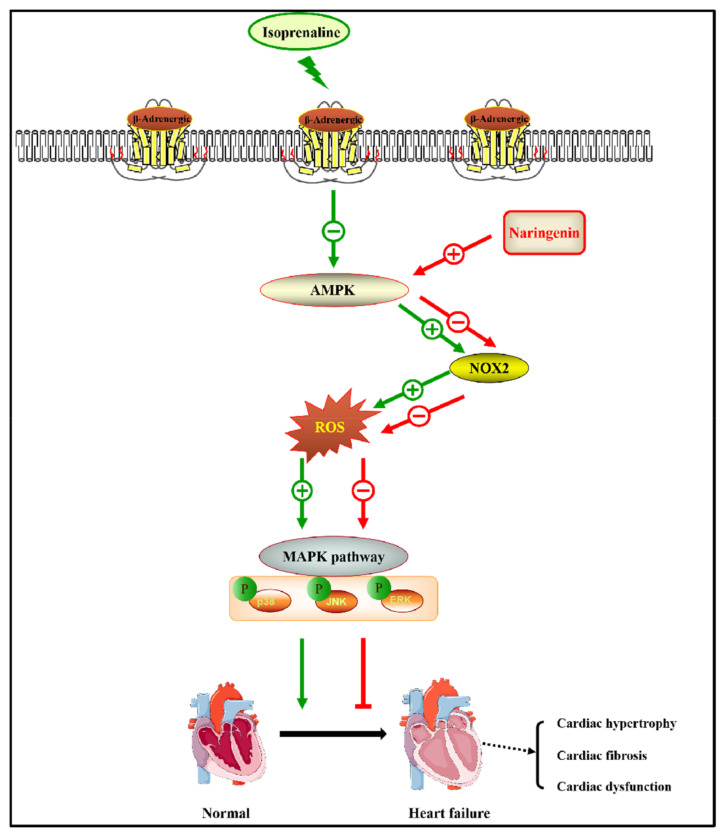
A schematic graph of the mechanisms of naringenin attenuating ISO-induced cardiac hypertrophy through suppressing oxidative stress via activating AMPK and inhibiting NOX2 and MAPK pathway.

The excessive ROS derived from NADPH oxidases activates various hypertrophy-related signaling pathways, for example, the MAPK signaling pathway [43]. Treatment with ISO has been reported to activate the MAPK signaling pathway [44], composed of P38, JNK, and ERK. The signaling pathway regulates a variety of biological behaviors, including cell proliferation, differentiation, and apoptosis [45]. The administration of ISO caused significant cardiac dysfunction in mice by activating MAPK-dependent oxidative stress [46]. Consistent with the reported results, our results show that ISO administration significantly upregulated the phosphorylation of MAPK-related signaling molecules (P38, JNK, and ERK) in heart tissues and NRCMs. Naringenin has been reported to prevent the activation of the MAPK signaling pathway, reduce oxidative stress, and protect the human bronchial epithelium from LPS-induced injury [47]. As with the previously reported results, we found that the pretreatment with naringenin reduced the phosphorylation of P38, JNK, and ERK. Meanwhile, the inhibition of AMPK by compound C mostly eliminated the inhibitory effect of naringenin on the MAPK signaling pathway, suggesting that naringenin exhibits an inhibition effect on cardiac hypertrophy via the MAPK signaling pathway (Figure 5).

Several clinical trials provided bioactivity, tolerability, and safety data of naringenin [48]. Even after daily naringenin-enriched food intake (i.e., whole orange juice 400 mL to 1000 mL), the serum naringenin concentration is far lower than the minimal effective concentration 8 μM [49], implying that naringenin obtained from daily food is insufficient, and additional naringenin supplementation is necessary to increase its serum concentration and enhance its biological role in the body. We also noticed that there is a report showing that that 4-week oral administration of 100 mg naringenin twice a day significantly reduced the body mass index, visceral fat level, and systolic blood pressure [50]. With regard to its safety, a single ingested dose of 900 mg of naringenin capsules is still safe in humans [48]. These data identified the efficiency and safety of naringenin. However, more clinical trials are needed to promote the translation of naringenin to clinical practice.

## 5. Conclusions

The present study suggested that pretreatment with naringenin attenuates ISO-induced cardiac hypertrophy. Additionally, the anti-hypertrophic effect of naringenin is mediated by inhibiting oxidative stress through the AMPK/NOX2/MAPK signaling pathway. Our results suggest an essential role of naringenin supplementation in treating myocardial hypertrophy. More studies are needed to confirm the effectiveness of naringenin in more animal models of cardiovascular disease. The encouraging results from animal experiments could promote naringenin into clinical trials as early as possible. Naringenin supplementation provides a new option for preventing and treating cardiovascular disease.

## Data Availability

Not applicable.

## Supplementary Materials

The following supporting information can be downloaded at: https://www.mdpi.com/article/10.3390/nu15061340/s1, Figure S1: Scheme of the experimental design; Figure S2: Cell viability assessed at different concentrations of naringenin with CCK8 kit; Table S1: The primer sequences of genes.
