# Supplementary material for: Naringenin Attenuates Isoprenaline-Induced Cardiac Hypertrophy by Suppressing Oxidative Stress through the AMPK/NOX2/MAPK Signaling Pathway
Source: Nutrients. 2023 Mar 9;15(6):1340. doi: 10.3390/nu15061340 (PMC10056776; doi:10.3390/nu15061340)
Supplement: Supplementary file 1 [file nutrients-15-01340-s001.zip › nutrients-2236476-supplementary.pdf]

Supplementary Materials:

Supplemental Figure S1: Scheme of the experimental design.

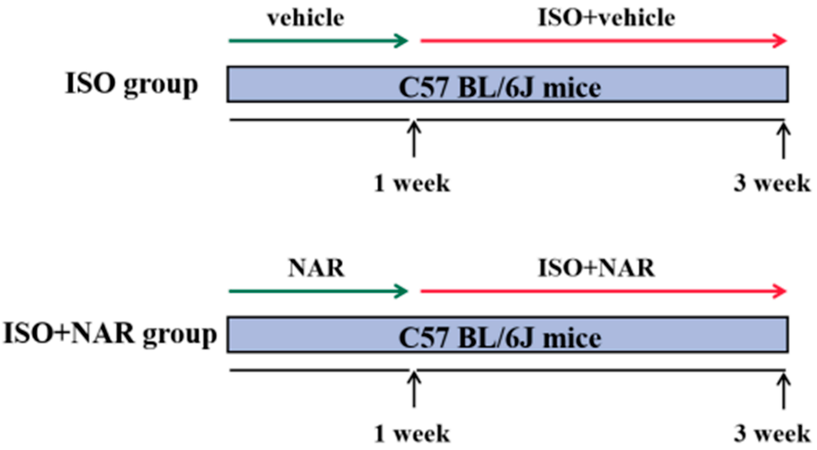

Supplemental Figure S2: Cell viability assessed at different concentrations of naringenin with CCK8 kit.

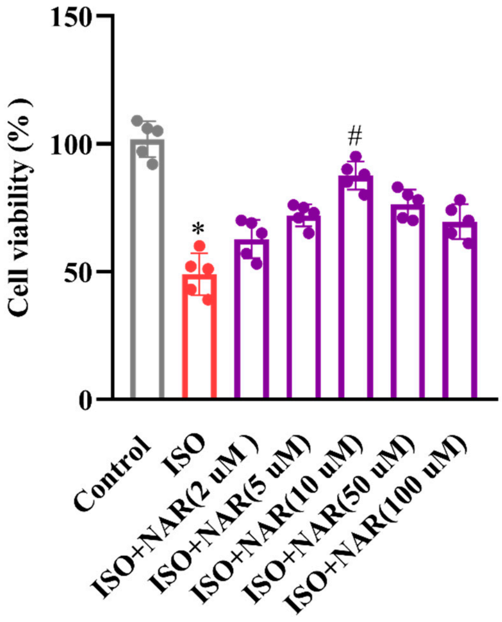

n=5, *P<0.05 versus Control group and #P<0.05 versus ISO group.

Supplemental Table S1: The primer sequences of genes.

Gene	5'-3' sequence
ANP (mouse)	Forward:TACAGTGCGGTGTCCAACACAG<br>Reverse: TGCTTCCTCAGTCTGCTCACTC
ANP (rat)	Forward:ATTGACAGGATTGGAGCCCA<br>Reverse:CAGAGTGGGAGAGGTAAGGC
BNP (mouse)	Forward:TCCTAGCCAGTCTCCAGAGCAA<br>Reverse: GGTCCTTCAAGAGCTGTCTCTG

BNP (rat)	Forward:CAGAAGGTGCTGCCCCAGATG<br>Reverse:GACTGCGCCGATCCGGTC
β-MHC (mouse)	Forward:GCTGGAAGATGAGTGCTCAGAG<br>Reverse:TCCAAACCAGCCATCTCCTCTG
β-MHC (rat)	Forward:TTTGATGTGCTGGGCTTCAC<br>Reverse:TGACATACTCGTTGCCCACT
NOX2 (mouse)	Forward:TGGCGATCTCAGCAAAAGGTGG<br>Reverse:GTACTGTCCCACCTCCATCTTG
NOX2 (rat)	Forward:CTGTGATAAGCAGGAGTTCCAA<br>Reverse:CCTGCACAGCCAGTAGAAGT
GAPDH (mouse)	Forward:CATCACTGCCACCCAGAAGACTG<br>Reverse:ATGCCAGTGAGCTTCCCGTTCAG
